# Retraction Note: The microRNA-130a-5p/RUNX2/STK32A network modulates tumor invasive and metastatic potential in non-small cell lung cancer

**DOI:** 10.1186/s12885-025-14700-0

**Published:** 2025-07-22

**Authors:** Fang Ma, Yangchun Xie, Yiyu Lei, Zengshuyu Kuang, Xianling Liu

**Affiliations:** 1 Department of Oncology, the Second Xiangya Hospital of Central South University, No. 139, Renmin Middle Road, Furong District, Changsha, 410000 Hunan P.R. China; 2 Department of Oncology, Zhuzhou 331 Hospital, Zhuzhou, 412000 Hunan P.R. China

**Retraction Note: BMC Cancer 20, 580 (2020)**

**https://doi.org/10.1186/s12885-020-07056-0**

The Editor has retracted this article because of significant concerns regarding a number of Figures presented in this work, which question the integrity of the data. After publication, overlap was detected with figures in articles submitted and published within a close time frame. Specifically, some panels from Fig. 2b appear to have been published in two other articles ([1, 2], now both retracted) and panels from Fig. 6b appear to have also been published in an article by other authors ([3], now retracted).

The authors stated that the figures were obtained through a commercial lab hired to perform some of the experiments for this study. Furthermore, authors stated that NSCLC cell lines A549, H1650 and SK-MES-1 might have been contaminated with HeLa cells.

The Editor therefore no longer has confidence in the integrity of the data in this article.

Dr. Fang Ma and Dr. Xianling Liu agrees with this retraction and none of the other authors have responded to correspondence from the Publisher about this retraction.
